# The Health-Seeking Behavior among Malaysian Adults in Urban and Rural Areas Who Reported Sickness: Findings from the National Health and Morbidity Survey (NHMS) 2019

**DOI:** 10.3390/ijerph19063193

**Published:** 2022-03-08

**Authors:** Sarah Nurain Mohd Noh, Suhana Jawahir, Yeung R’ong Tan, Iqbal Ab Rahim, Ee Hong Tan

**Affiliations:** 1 Centre for Health Equity Research, Institute for Health Systems Research, Ministry of Health Malaysia, Shah Alam 40170, Malaysia; suhana.j@moh.gov.my (S.J.); dr.tanyr@moh.gov.my (Y.R.T.); fathullah@moh.gov.my (I.A.R.); jdreehong@moh.gov.my (E.H.T.); 2 National Institutes of Health, Ministry of Health Malaysia, Shah Alam 40170, Malaysia; 3 Melaka State Health Department, Ministry of Health Malaysia, Ayer Keroh 75450, Malaysia

**Keywords:** health-seeking behavior, urban population, rural population, adult, self-medication, healthcare provider, Malaysia

## Abstract

Understanding care-seeking behavior among urban and rural populations can help to support the planning and implementation of appropriate measures to improve health in the community. This study aims to determine the factors associated with the health-seeking behavior among Malaysian adults in urban and rural areas who reported sickness. This study used data of Malaysian adults aged 18 years and over from the National Health and Morbidity Survey 2019; a cross-sectional, national household survey that targeted all non-institutionalized residents in Malaysia. Respondent’s characteristics and health-seeking behavior were described using complex sample descriptive statistics. Multivariable logistic regression analysis was conducted to examine the association between potential factors (sociodemographic characteristics, enabling, and health need) and health-seeking behaviors (seeking treatment from healthcare practitioners and self-medication). A total of 10,484 respondents, estimated to represent 18.9 million Malaysian adults aged 18 years and over, were included in the analysis. Prevalence of seeking treatment from healthcare practitioners and self-medication among Malaysian adults with self-reported sickness were 57.3% and 23.3%, respectively. Self-reported sickness among both the urban and rural populations who rated their health as poor to very poor was more likely to seek treatment than those who rated good to excellent. However, among the urban population, those who rated their health as poor to very poor were less likely to self-medicate. Among the urban population, government employees were more likely to seek treatment, and being without formal education significantly increased the likelihood to self-medicate. Among the rural population, those with at least one long-term condition were more likely to seek treatment than those with none. Understanding the factors which influence health-seeking behavior among the urban and rural population could close the gaps in healthcare utilization among the population in Malaysia.

## 1. Introduction

Health-seeking behavior is one of the major determinants of health outcomes in a community. It determines how health services are used which influences population health outcomes [1]. Health or care-seeking behavior is defined as any action undertaken by individuals who perceive themselves to have a health problem or to be ill to find an appropriate remedy [2]. Individual attributes, the essence of the community in which a person resides, and the relationship between individual and environmental factors are all linked to the health-seeking behavior of an individual [3]. 

There has been a growing interest in research related to health-seeking behaviors over the years locally and internationally [4,5]. Studies conducted locally among the urban population found that 63.5% of participants used self-medication for minor ailments [6], 85% consumed over-the-counter (OTC) medications [7], while 67% chose to consult the physician when they experienced any health problems [8]. A national study in Brazil indicated that the prevalence of use of medicine via self-medication was 18.3% [9]. Another study among the rural and urban population of Karachi, Pakistan reported that 93% of the respondents had practiced self-medication [10].

Several theoretical models explain health behaviors; Evans and Stoddart Model [11], Health Belief Model [12], Grossman Model of Health Demand [13], and Andersen’s Behavioral Model [14]. Andersen’s Behavioral Model of Health Care Utilization is one of the most widely used for predicting health-seeking behaviors due to the convenience of application and popularity in modeling studies involving healthcare accessibility and utilization [14].

Geographic location has a significant influence on the accessibility to healthcare services [15,16], and access to healthcare is reported as one of the many pivotal factors contributing to the gap in health equity among the urban and rural populations [17]. The Malaysian health system is based on a geographically widespread healthcare delivery system designed to provide the entire population with access to public health services, both in the rural and urban localities [18]. An urban area in Malaysia is classified as a gazetted area with a combined population of 10,000 or more, whereas a rural area is defined as a gazetted area with a combined population of less than 10,000 [19]. The equitable healthcare financing and structured public healthcare system in Malaysia [20] does not inherently translate to equitable access because geographical barriers exist [21], among other factors. 

In the health sector, access and utilization are interrelated concepts, with access playing a critical role in the utilization of healthcare services. Access to health care was defined as “actual use of personal health services and everything that facilitates or impedes their use” [22]. According to Levesque’s Conceptual Framework of Access to Health, the five dimensions of accessibility are approachability, acceptability, availability/accommodation, affordability, and appropriateness [23]. Internationally, research has documented differences in access to and utilization of health care services between urban and rural populations, which consequently affected their health outcomes. Rural patients experienced more barriers to access health care (i.e., distance, travel time, transportation, infrastructure building, medical resources, staff distribution, and clinic distribution) as compared to their urban counterparts [18,24,25,26,27,28,29], which resulted in having to restart the care-seeking process, inappropriate use of emergency departments, unmet need for care, or health problem exacerbation [25,30].

The introduction of the New Economic Policy in the 1970s has increased the urbanization rate from 26.8% in 1970 to 71.0% in 2010, which were expected to rise to 76.6% in 2020 and 88.0% in 2050 [31]. As of 2020, the Malaysian public healthcare system has a distribution of 3171 clinics and 154 hospitals throughout the country, which also provide mobile clinic services to remote areas. There were 7988 registered clinics and a total of 250 licensed hospitals, maternity homes, nursing homes, and hospices in the private healthcare facilities in Malaysia, which are mostly concentrated in the urban areas [18,32,33]. The allocation of healthcare services and resources within the public sector was uneven, favoring urban clinics heavily [24]. Compared to rural areas, urban areas have a greater density of primary care clinics and health workers per capita (2.2 clinics and 15.1 healthcare practitioners per 10,000 population in urban areas versus 1.1 clinics and 11.7 healthcare practitioners per 10,000 population in rural areas) [24]. 

Malaysia has a dual healthcare system, where the main providers of healthcare are public and private sectors [34]. In ensuring efficiency through decentralization, the hierarchical organization structure of the Ministry of Health (MOH) Malaysia is stratified into the Federal, State, and District levels [35]. Funded through general revenue, the public sector aims to provide universal access with a focus on low-cost but high-benefit health care programs. To keep up with the population growth, especially in urban areas, the dual healthcare system has developed with the private sector serving mostly urban regions and better-off patients with fee-for-service primary and secondary care, while the public sector maintains its social equity mission, including primary care services for poor and rural populations [34]. As an expansion of healthcare services in Malaysia, pharmacy practice has also evolved beyond traditional dispensing, from a product-oriented to patient-oriented service where in-house pharmacists provided counseling in drug safety, poison information, and medication understanding. Some community pharmacies offered other services such as blood pressure monitoring, chronic disease screening [36], and weight management [37,38]. Although the expansion of community pharmacies in Malaysia means people may have more access to over-the-counter medicine, the MOH has implemented rules about prescription-only medicines, such as antibiotics. Other studies found that the causes of misuse and overuse leading to antibiotic resistance are various [39], and largely due to antibiotics dispensed without a prescription [40]. National guidelines on antibiotics have also been made accessible to the public and healthcare practitioners [41,42].

The duality of Malaysia’s healthcare system is further magnified with the practice of both conventional Western medicine (also referred to as modern medicine) as well as traditional and complementary medicine (T&CM) as part of its healthcare services [18,43,44]. Under the enforcement of the T&CM Act, T&CM such as herbal therapy, acupuncture, and traditional massages were also incorporated in some public & private hospitals as supplementary treatment modalities [43,45]. This was in line with the World Health Organization’s efforts to maximize the potentials of safe and quality T&CM services as a complement to modern medicine among its member states, to achieve holistic healthcare as part of the Universal Health Coverage (UHC) initiative [43,46,47].

Understanding health-seeking behavior and its associated factors would enable health systems to review strategies to accommodate healthcare expectations in the community [48]. Although this knowledge is vital in the proper designing of healthcare policies, very few studies have been conducted at the national level to explore the factors which influence health-seeking behavior among the adult population in the urban and rural areas in Malaysia. In this study, we aim to (1) determine the characteristics of respondents based on locality (urban-rural), (2) determine the prevalence of sick Malaysian adults based on locality, and (3) determine the factors associated with the health-seeking behavior of Malaysian adults who reported sickness, according to locality.

## 2. Materials and Methods

### 2.1. Study Design and Participants

The data for this study was obtained from the National Health and Morbidity Survey (NHMS) 2019, a cross-sectional household survey with a two-stage stratified sampling method to ensure national representativeness. It was conducted among the population in Malaysia who were non-institutionalized and residing in the selected households for at least 2 weeks before the data collection. States and federal territories constituted the primary stratum, and urban and rural areas within the states were considered the secondary stratum. The sampling frame for this survey was provided by the Department of Statistics Malaysia using the National Population and Housing Census 2010. All 13 states and 3 federal territories were included in this survey. Within each state, the required number of Enumeration Blocks (EBs) from urban and rural areas were randomly chosen. First stage sampling involved a random selection of 463 EBs (350 urban and 113 rural) from the total EBs in Malaysia (over 75,000 EBs) via a probability proportional to size sampling technique. Subsequently, in each selected EB, 14 Living Quarters (LQs) were selected during the second-stage sampling. All households within the selected LQs and all members in the households were invited to participate in this survey. A total of 5365 LQs were successfully visited giving an LQ response rate of 92.6% and a total of 16,688 respondents were successfully interviewed giving an individual response rate of 90.0%. The overall response rate for this community-based survey was therefore 83.4%. A detailed methodology and sampling design of the survey is described in the NHMS 2019 official report [44].

A total of 10,933 Malaysian adults aged 18 years and over participated in the survey. Only data of respondents with complete responses on potential predictors (sociodemographic characteristics, enabling, and health need factors), experienced acute health problems, and health-seeking behaviors (seeking treatment from a healthcare practitioner and self-medication) were included in this study. In this study, the proportion of missing data was 4.11% (*n* = 449) and the missing data proportion of less than 5% was acceptable for complete case analysis [49]. When a preliminary analysis of all respondents was conducted, including those with missing data, no differences in results were observed.

### 2.2. Data Collection

In NHMS 2019, data were collected from July to October 2019 by trained research assistants, via face-to-face interviews using a validated questionnaire [50,51]. The questionnaire was programmed into an application and uploaded onto digital tablets as mobile data collection tools. The tablets were used to collect data, store and back up data in the SD cards, and upload data to the central system. To ensure the minimum sample size required is achieved, vacant or closed houses during the first visit were revisited up to at least three times. The tenets of the Declaration of Helsinki were followed during the study. Written informed consent was obtained from all participants before the interviews. The Medical Research and Ethics Committee (MREC), MOH Malaysia granted permission to carry out the National Health and Morbidity Survey 2019 (NMRR-18-3085-44207).

### 2.3. Study Variables

#### 2.3.1. Andersen’s Behavioral Model of Health Care Utilization

Andersen’s Behavioral Model of Health Care Utilization was adapted into this study for its convenience of application and popularity in modeling studies involving healthcare accessibility and utilization. The model suggests that the health-seeking behavior of individuals is influenced by three groups of factors: sociodemographic characteristics, enabling, and health needs. Sociodemographic characteristics describe the tendency to use the services (i.e., sex, ethnicity, age, education level, and marital status), enabling factors describes the resources available to use the health services and facilities (i.e., wealth status, social support, and access to health resources), and health need factors represent perceived need for healthcare services [14].

#### 2.3.2. Dependent Variables

In this study, there are two dependent variables included which are: (1) seeking treatment from healthcare practitioners among those who reported sickness for the last 2 weeks before the interview, and (2) self-medication among those who reported sickness for the last 2 weeks before the interview. Those who reported sickness in the last 2 weeks before the interview were respondents who answered “yes” to the question “In the last 2 weeks, did you experience any of the following health problems such as fever, sore throat, difficulty in swallowing, running nose or blocked nose, cough, and others”

Those who answered “yes”, were then asked their health-seeking behavior (yes or no) based on the question “In the last 2 weeks, did you seek treatment/medication or advice from healthcare practitioners?” and “In the last 2 weeks, did you take medicine without advice from healthcare practitioners?” In this study, the term seeks treatment was used to refer to “seek treatment/medication or advice from healthcare practitioners” in short. Healthcare practitioners refer to modern healthcare practitioners including community pharmacists as well as traditional and complementary medicine practitioners (e.g., spiritual healer, Chinese herbalist, Ayurvedic practitioner, and Islamic medicine practitioner). Self-medication was used to refer to “take medicine without advice from healthcare practitioners”

#### 2.3.3. Independent Variables

##### Sociodemographic Characteristics

In this study, sociodemographic variables included were: sex (male or female); ethnicity (Malay or Non-Malay); age (a continuous variable, grouped into 18–34, 35–59, or 60+ years); education level (no formal education, primary, secondary, or tertiary education); and marital status (single, married, or widow(er)/divorced/separated). The age of respondents in years was grouped into “18–34”, “35–59”, and “60+ years” based on age distribution pattern. Education levels were categorized into four groups: no formal education, primary, secondary, and tertiary education. Respondents who had never been to school to get any form of education or did not complete primary school were categorized into ‘no formal education, while those who completed Standard Six were categorized as ‘primary’ education level. ‘Secondary’ education level represented those with at least five years of schooling at secondary school, whereas ‘tertiary’ education level represented those who completed Form Six or received certificates, diplomas, or academic degrees.

##### Enabling Factors

The enabling factors included were: employment status (government employee, private employee, self-employed, or unemployed); income (quintile 1 (Q1), quintile 2 (Q2), quintile 3 (Q3), quintile 4 (Q4), or quintile 5 (Q5)), calculated based on total monthly household income and then were grouped into quintiles; and healthcare coverage (yes or no). Q1 represents the poorest 20% of the population and Q5, the 20% richest. Healthcare coverage was defined as having supplementary financial coverage for health care such as government employees’ health benefits, pensioner cards, government-specific health fund, personal health insurance, employer-sponsored insurance, and panel clinic.

##### Health Need Factors

Proxy measures for health needs included were: self-rated health (good to excellent, fair, or poor to very poor); and presence of at least one long-term condition (yes or no), assessed from the questions “Have you ever been told by a doctor or assistant medical officer that you have diabetes?”, “Have you ever been told by a doctor or assistant medical officer that you have high blood pressure?” and “Have you ever been told by a doctor or assistant medical officer that you have high cholesterol?” For the analysis, respondents who answered at least one “yes” to either one of the conditions, were coded as “yes” to “presence of at least one long-term condition”

### 2.4. Statistical Analysis

Secondary data analysis was conducted using STATA version 14 (Stata Corp, College Station, TX, USA). Complex sample descriptive statistics were used to illustrate the sociodemographic, enabling, and health need characteristics of the respondents, according to their locality (urban-rural). Sample weights and study design were taken into consideration using a complex sampling design in all data analyses. The products of the inverse of the probability of sampling, a non-response adjustment factor, and a post-stratification adjustment by age, gender, and ethnicity were used to calculate the weight used for estimation.

Comparison of characteristics between urban and rural populations was performed using the chi-square test. Univariate with the chi-square test and multivariable logistic regression analysis, which presented as crude odds ratios (COR) and adjusted odds ratios (AOR) with 95% confidence intervals (CI), were used to predict characteristics of those who sought treatment from healthcare practitioners, and those who self-medicated, stratified by urban-rural locality. All variables with a *p*-value < 0.25 in the univariate analysis were considered as predictive variables and entered into multivariable regression analysis [52]. The multivariable analysis was performed for urban and rural separately to examine the predictive factors for seeking treatment and self-medication using four models while adjusting for all other potential covariates such as sociodemographic characteristics, enabling, and health need factors. The AOR with a 95% confidence interval was determined where *p*-value < 0.05 was considered statistically significant. The goodness of fit model was tested using Hosmer–Lemeshow statistics, and *p*-value > 0.05 was considered as a good fit.

## 3. Results

A total of 10,484 respondents representing 18.9 million population were included in the analysis. The respondents comprised of urban population (76.1%) and rural population (23.9%). Table 1 shows the sociodemographic characteristics, enabling, and health need factors of the respondents, stratified by locality. Both urban and rural populations had significant differences in all factors, except marital status and the presence of at least one long-term condition.

Table 2 presents the prevalence of Malaysian adults who reported sickness. The overall prevalence of Malaysian adults who reported sickness was 16.1%. Of these, more than half (57.3%) sought treatment from healthcare practitioners, and about a quarter (23.3%) self-medicated. The prevalence of Malaysian adults in the rural areas who reported sickness (17.6%) was higher than the urban adults (15.6%). There were significant differences in the prevalence of those who reported sickness by different sociodemographic characteristics. Among the urban population, a higher prevalence of self-reported sickness was seen among females. Among the rural population, a higher prevalence of self-reported sickness was seen among non-Malays, aged 60 and over, those without formal education as well as a widow(er)/divorced/separated. Prevalence of self-reported sickness among those who self-rated their health as poor to very poor and those with at least one long-term condition was higher among both the urban and rural populations. Among those who reported sickness, more than half (57.3%) sought treatment from healthcare practitioners, and about a quarter (23.3%) self-medicated.

Table 3 displays the results of the logistic regression analysis of health-seeking behaviors, with COR and AOR, and their CIs and *p*-values. The model I and II assessed the factors associated with seeking treatment among self-reported sick adults in urban and rural localities, respectively. The multivariable logistic regression revealed that employment status and self-rated health were significantly positively associated with seeking treatment among the urban population. Among urban dwellers, government employees were about 2 times (AOR = 1.82, 95% CI: 1.01–3.27) more likely to seek treatment than those who were self-employed. Urban dwellers who rated their health as poor to very poor were about 3 times (AOR = 2.94, 95% CI: 1.47–5.88) more likely to seek treatment than those who rated good to excellent. 

Whereas among the rural population, self-rated health and presence of any long-term conditions were significantly positively associated with seeking treatment. Urban dwellers who rated their health as poor to very poor were about 4 times (AOR = 3.68, 95% CI: 1.36–9.97) more likely to seek treatment than those who rated good to excellent health, whereas those with at least one long-term condition were about 2 times (AOR = 2.06, 95% CI: 1.23–3.45) more likely to seek treatment than those with none. 

Model III and IV assessed the factors associated with self-medication among self-reported sick adults in urban and rural localities, respectively. The regression revealed that education levels were significantly positively associated with self-medication among urban dwellers, where being without formal education significantly increased the likelihood of about 4.3 times (AOR = 4.29, 95% CI: 1.81–10.17) to self-medicate. Whereas self-rated health was significantly negatively associated with self-medication among the urban population. Urban dwellers who rated their health as poor to very poor were less likely (AOR = 0.40, 95% CI: 0.16–0.98) to self-medicate than those who rated good to excellent. However, in terms of self-medication among those who reported sickness in the rural locality, there was no significant association found. The Hosmer–Lemeshow test showed the goodness-fit of the models (*p* > 0.05). Thus, these models were considered a good fit.

## 4. Discussion

This study aimed to determine the characteristics of respondents and prevalence of Malaysian adults who reported sickness based on their urban-rural locality, as well as the factors associated with their health-seeking behaviors. All variables, excluding marital status and the presence of at least one non-communicable disease, were substantially different between the urban and rural populations. The overall prevalence of Malaysian adults who reported sickness was 16.1%, and higher among the rural population as compared to the urban population. Higher prevalence of self-reported sickness among those who self-rated their health as poor to very poor and those with at least one long-term condition were seen among both the urban and rural populations. More than half of those who reported sickness sought treatment from healthcare practitioners, while only about a quarter self-medicated. Self-rated health was one of the factors associated with health-seeking behavior among Malaysian adults who reported sickness from the urban and rural areas.

Overall, less than a fifth of Malaysian adults reported sickness, with the rural populations exhibiting a higher prevalence than those from the urban areas. Similarly, other published studies found that illnesses were more prevalent among the rural population [27,28,53]. As an upper-middle-income country, Malaysia’s population has benefited from a well-developed health care system, together with improved access to clean water, sanitation, and better child nutrition, which was reinforced through programmes targeted at reducing poverty, increasing literacy, and providing modern infrastructure [54], and these developments may have an effect on the overall population health. Compared to Denmark (about 9 out of 10 respondents reported having experienced at least one symptom) [55] and Hong Kong (46.5% of the respondents aged between 16 and 54 years reported having any symptoms) [56], Malaysia’s population had a better health status in terms of overall prevalence of reported recent illnesses. However, owing to variations in methodology and variables evaluated, these results are not directly comparable.

More than half of those who reported sickness (58.9% of urban and 52.6% of rural) sought treatment from healthcare practitioners in the current study and the prevalence was lower among the rural population. Seeking treatment from healthcare practitioners was the first choice of health-seeking behavior reported by previously published studies [29,57]. However, given our results suggest that only slightly more than half of the population sought medical attention, this raises concerns about the proportion of people who did not seek appropriate treatment or care. A study conducted locally reported that 4.9% and 5.4% of urban and rural participants, respectively, did not seek treatment when they were sick [29]. Low perception of illness as a major health problem [44,58], low perceived need to seek care [59], work commitment [44], financial constraint [3,44,59,60], and geographical locale [61] were barriers reported in previous studies. As health needs and challenges have changed over the past decade, policymakers must consider the factors that influence people’s health-seeking behavior. For the sustainable and equitable provision of health care to the disadvantaged and underserved groups, removing barriers and integrating public and private health services are crucial [62].

Malaysia is among the countries that have achieved UHC, with the vast majority of the population receiving comprehensive public healthcare services [63]. Malaysia, like most other countries, has a two-tiered healthcare system, with a highly subsidized public sector and a fee-for-service private sector [64]. However, this study findings showed that sick rural adults were less likely than their urban counterparts to seek healthcare from a healthcare practitioner. While most studies from other countries have identified socio-cultural norms as determinants, distance and proximity to a healthcare facility also was identified as a significant factor for this behavior [65,66,67,68]. Within the public sector, the distribution of healthcare facilities and resources heavily favored urban areas [20,25,26,27,28,29,31]. Furthermore, the current study found that a larger percentage of people in rural areas fall into the lower income quintiles. Inadequate access to health care and a lack of income are two reported factors that contribute to the rural population’s poor health [65,66,67,68]. As the majority of Malaysians with low socioeconomic status came from rural areas [69], this calls for more efforts to promote healthcare utilization and enhance accessibility in the remote and rural areas.

This study found that less than a fifth of the population who reported sickness practiced self-medication, which was lower than previous population-based studies [27,28] as well as other local studies [70,71,72], but higher in study conducted in Sri Lanka (Urban: 12.2%, Rural: 7.9%) [73]. This could be because self-medication in Malaysia is more costly compared to seeking treatment from healthcare practitioners, as patients are only charged minimal fee of Malaysian Ringgit (MYR1) [US dollars (USD0.24)] for visits to the public health clinics [74]. Although self-medication assists in the reduction of the burden on medical care, it is linked to many possible risks [70,75,76,77]. This issue highlights the importance of healthcare practitioners in promoting rational use of medicines, including information on potential side effects, ensuring informed and responsible self-medication [77]. Moreover, public health awareness programmes can be organized as part of larger public health efforts, to help people understand disease processes and positive health behaviors.

According to the World Health Organization, education is one of the key social determinants of health, and addressing it appropriately is essential to promote health and reduce long-standing health inequities [78]. Among the urban population in our study, those with no formal education were more likely to self-medicate than those with higher education levels. The influence of education level on self-medication practice is consistent with a study in Saudi Arabia [79]. In Malaysia, community pharmacies that are strategically and conveniently located in shopping malls and supermarkets [33] led to better access to OTC medications, especially among the urban population where amenities and infrastructure are more readily available. While OTC medications have been shown to be safe and appropriate for use without the supervision of a health care provider, unwanted effects may result if used irresponsibly [80]. Inadequate health literacy among the less educated coupled with easy access to medication may result in serious consequences, which prompts the need to improve health literacy, particularly the negative consequences of self-medication to one’s wellbeing. Furthermore, people with a lower degree of education usually have lower health literacy [81]. Thus, the combined effect of easy access to the medications and the higher likelihood of self-medication among those with the lowest educational attainment of the urban population posed a worrying situation. Campaigns such as ‘Know Your Medicines’, in line with Malaysia’s national health agenda, ‘*Agenda Nasional Malaysia Sihat*’ (ANMS), advocate the importance of knowing your medications to improve public awareness and empower personal health [82].

Results from our study indicated that self-rated health to be one of the important associated factors in health-seeking behavior. Those who self-rated their health as poor to very poor were more likely to seek care than those who self-rated their health as good to excellent, regardless of locality. Conversely, those who self-rated poor to very poor health was also less likely to self-medicate than those who self-rated good to excellent health among urban population. Published literatures highlighted the association of health-enhancing behaviors, utilization of health services [83,84], and self-medication [79], among those who rated their health as poor, however, the reported results are mixed. Previous studies have established that a single-item measure of self-rated health provides a holistic view of the population’s physical and emotional well-being, as well as the ability to predict health-seeking behavior and healthcare use [50,83,85]. In our study, those who self-rated their health as poor to very poor were significantly associated with the presence of long-term condition(s) (Table S1), which is consistent with another large-scale study conducted in China [86]. The relationship between presence of long-term condition(s) and poorer health could explain the influence of self-rated health on health-seeking behaviors as the nature of the long-term condition itself, which demands follow-up appointments and a formal prescription to obtain the medications, that may cause this group of people used the health care services and less likely to self-medicate. 

Among the urban population, government employees were more likely to seek treatment than those who were self-employed when they were sick. The association between occupational status and treatment seeking behavior in this study result is consistent with another study conducted in China, which found that self-employed people were less likely to take remedial action and seek medical help after being ill [87]. This could be attributed to the time constraints to seek treatment when they are sick and financial issues, as self-employed individuals were more likely to have irregular working hours. Furthermore, their incomes are directly dependent on their work [88]. Additionally, government employees are entitled to a higher number of days for paid sick leave [89] compared to those working in other than the public sector [90].

Rural population with at least one long-term condition were more likely to seek medical treatment than those without, which concurred with previous research that found an association between the presence of chronic illnesses and seeking healthcare services [50,91]. Two-thirds of public primary care clinics in Malaysia are in rural areas [24] and a national cross-sectional study of randomly selected clinics found that doctors in public clinics saw more chronic diseases like hypertension and diabetes, as well as follow-up cases, whereas doctors in private clinics saw more acute and minor illnesses [92]. This occurrence may largely be contributed by the heavily subsidized public healthcare services by the Malaysian government, that also covers the cost of lifelong medications, which is more economical for patients with chronic disease as the nominal fee granted access to the entire spectrum of public healthcare services in the clinics [18,21,64,74,92]. This economic factor may have driven private clinics away from the rural areas [18,24]. Perceived severity or fear of the consequences of the disease [93] might also be the reason for seeking treatment among the rural population.

This study discovered that gender is not associated with health-seeking behavior among Malaysian adults who reported sickness. Although women were perceived more likely to seek treatment and utilize health services as compared to men [5], previous local studies found that, in general, there was no difference in terms of healthcare utilization across gender [94,95,96]. In addition, previous national health survey reported that there was no difference in the autonomy of decision making for healthcare between gender [28].

The sample size for this study was large, consisting of 10,484 adults who covered both the urban and rural areas. The proportion of respondents from the urban and rural areas in this survey was very close to Malaysia’s real population in the same year [97]. Despite its strengths, this research has a number of limitations. Because of the cross-sectional nature of this research, no causal association between health-seeking behavior and associated factors could be established. Seasonal change could not be measured as the data was collected at just one point in time. Finally, since this analysis used self-reported data on previous events, there is a possibility of recall bias.

## 5. Conclusions

This cross-sectional study showed that sociodemographic, enabling, and health need characteristics were associated with health-seeking behaviors among Malaysian adults who reported sickness from both urban and rural localities, with education level, employment status, self-rated health, and presence of at least one long-term condition as the associated factors. This study revealed gaps in healthcare services and more rooms for improvements despite Malaysia has already achieved UHC status. Understanding the factors which influence health-seeking behavior among the urban and rural population could close the gaps in healthcare utilization among the Malaysian population. Future policies should move towards specific targeted approaches that focus on the rural and vulnerable population, especially regarding access to healthcare services as well as their knowledge and literacy on seeking proper medical care.

Taking care of health should be a culture, a way of life. It should be embedded and be a shared responsibility across all sectors, in line with the Sustainable Development Goals. Social services actors and organizations, which administratively are not under the purview of the MOH Malaysia, are closer to the people’s hearts as compared to governmental organizations. Political players are the main drivers with powers to influence the masses. Mainstream and social media are also key players in educating the nation regarding health matters. We recommend active two-way engagements, dialogues, and close collaborative efforts with these parties for a shared vision of a healthy nation. We also recommend further in-depth studies to be conducted on factors such as perceived quality of services received, which may provide a deeper understanding on the health-seeking behavior of Malaysia population.

## Figures and Tables

**Table 1:** Characteristics of respondents, stratified by urban-rural locality (N = 10,484).

Characteristics	Count, *n* (Unweighted)	Estimated Population, *n* (Weighted)	% Weighted (95% CI)	Locality		*p*-Value
				Urban	Rural	
				% Weighted (95% CI)	% Weighted (95% CI)	
***Sociodemographic***						
**Sex**						
Male	4905	9,116,299	48.2 (47.0–49.5)	49.8 (48.3–51.3)	43.2 (40.9–45.5)	<0.001 *
Female	5579	9,778,742	51.8 (50.5–53.0)	50.2 (48.7–51.7)	56.8 (54.5–59.1)	
**Ethnicity**						
Malay	7237	10,810,187	57.2 (52.9–61.4)	52.7 (47.5–57.9)	71.5 (65.8–76.5)	<0.001 *
Non-Malay	3247	8,084,854	42.8 (38.6–47.1)	47.3 (42.1–52.5)	28.5 (23.5–34.2)	
**Age (years)**						
18–34	3257	7,788,423	41.2 (39.7–42.7)	41.0 (39.2–42.9)	41.8 (39.4–44.1)	<0.001 *
35–59	4848	7,966,079	42.2 (40.7–43.6)	43.6 (41.9–45.3)	37.6 (35.4–39.8)	
60+	2379	3,140,539	16.6 (15.4–18.0)	15.4 (13.9–17.0)	20.6 (18.4–23.1)	
**Education level**						
No formal	526	704,841	3.7 (3.2–4.3)	2.7 (2.2–3.4)	7.0 (6.0–8.1)	<0.001 *
Primary	2179	3,285,633	17.4 (16.1–18.7)	14.7 (13.3–16.3)	25.8 (23.4–28.5)	
Secondary	5077	9,516,574	50.4 (48.7–52.1)	50.7 (48.6–52.8)	49.4 (46.8–52.0)	
Tertiary	2702	5,387,993	28.5 (26.6–30.5)	31.9 (29.5–34.4)	17.8 (15.2–20.6)	
**Marital status**						
Single	2213	5,349,399	28.3 (26.7–30.0)	28.6 (26.7–30.7)	27.3 (24.7–30.0)	0.147
Married	7116	11,932,813	63.2 (61.3–64.9)	63.3 (61.0–65.4)	62.8 (59.9–65.6)	
Widow(er)/Divorcee/Separated	1155	1,612,829	8.5 (7.7–9.4)	8.1 (7.7–9.4)	9.9 (8.6–11.4)	
***Enabling***						
**Employment status**						
Government	1177	1,511,788	8.0 (7.1–9.1)	8.2 (7.1–9.5)	7.4 (5.9–9.2)	<0.001 *
Private	2873	6,351,279	33.6 (31.7–35.6)	38.1 (35.8–40.6)	19.3 (16.6–22.3)	
Self-employed	1977	3,493,565	18.5 (17.2–19.9)	16.5 (15.0–18.1)	24.9 (22.4–27.5)	
Unemployed	4457	7,538,409	39.9 (38.3–41.5)	37.2 (45.4–51.5)	48.5 (45.4–51.5)	
**Household income quintile**						
Q1 (20% poorest)	2277	3,932,420	20.8 (19.3–22.4)	17.1 (15.5–18.9)	32.6 (29.2–36.3)	<0.001 *
Q2	1992	3,398,139	18.0 (16.4–19.7)	15.7 (13.9–17.7)	25.3 (22.4–28.4)	
Q3	2003	3,754,531	19.9 (17.9–22.0)	20.4 (18.0–23.0)	18.3 (15.7–21.3)	
Q4	2093	3,786,071	20.0 (18.2–22.0)	22.1 (19.9–24.6)	13.3 (10.9–16.2)	
Q5 (20% richest)	2119	4,023,880	21.3 (18.9–23.9)	24.7 (21.8–27.9)	10.5 (8.0–13.6)	
**Covered by any healthcare coverage**						
Yes	5609	10,439,085	55.2 (53.1–57.4)	60.6 (57.9–63.1)	38.4 (34.8–42.0)	<0.001*
No	4875	8,455,956	44.8 (42.6–46.9)	39.4 (36.9–42.1)	61.6 (58.0–65.2)	
*Health need*						
**Self-rated health**						
Excellent & Good	7856	14,814,257	78.4 (76.9–79.9)	80.0 (78.3–81.7)	73.2 (70.1–76.2)	<0.001 *
Fair	2371	3,689,380	19.5 (18.2–21.0)	17.9 (16.4–19.6)	24.7 (22.0–27.5)	
Poor & Very Poor	257	391,404	2.1 (1.7–2.5)	2.1 (1.7–2.6)	2.1 (1.5–2.8)	
**Presence of at least one long-term condition**						
Yes	3148	4,639,737	24.6 (23.3–25.8)	24.2 (22.7–25.7)	25.7 (24.0–27.5)	0.211
No	7336	14,255,304	75.4 (74.2–76.7)	75.8 (74.3–77.3)	74.3 (72.5–76.0)	

*n*, count; %, percentage; CI, Confidence Interval; Q, Quintile. *p*-values were obtained by chi-square test; * indicates a statistical significance. Healthcare coverage refers to government employees’ health benefits, pensioner cards, government-specific health funds, personal health insurance, employer-sponsored insurance, and panel clinic. Long-term condition refers to presence of any non-communicable diseases (diabetes, hypertension or hypercholesterolemia).

**Table 2:** Prevalence of those who reported sickness among Malaysian adults, stratified by urban-rural locality.

Characteristics	Overall (N = 10,484)		Urban (*n* = 6288)			Rural (*n* = 4196)		
	Count	% Weighted (95% CI)	Count	% Weighted (95% CI)	*p*-Value	Count	% Weighted (95% CI)	*p*-Value
OVERALL	1946	16.1 (14.8–17.4)	1187	15.6 (14.1–17.3)	-	759	17.6 (15.5–19.9)	-
								
***Sociodemographic***								
**Sex**								
Male	778	14.1 (12.6–15.7)	460	13.3 (11.7–15.2)	<0.001*	318	16.7 (14.1–19.7)	0.441
Female	1168	17.9 (16.2–19.8)	727	17.8 (15.8–20.1)		441	18.2 (15.5–21.3)	
**Ethnicity**								
Malay	1349	15.8 (14.3–17.5)	793	16.4 (14.5–18.5)	0.283	556	14.5 (12.2–17.1)	<0.001 *
Non-Malay	597	16.4 (14.3–18.8)	556	14.7 (12.4–17.3)		203	25.3 (21.0–30.3)	
**Age (years)**								
18–34	580	15.3 (13.5–17.3)	391	15.6 (13.4–18.1)	0.407	189	14.3 (11.4–17.8)	0.005 *
35–59	875	15.8 (14.2–17.6)	559	15.0 (13.1–17.0)		316	18.8 (15.8–22.2)	
60+	491	18.7 (16.4–21.4)	237	17.4 (14.4–20.9)		254	21.9 (18.5–25.8)	
**Education level**								
No formal	120	23.0 (18.1–28.8)	40	17.0 (10.8–25.8)	0.148	80	30.4 (23.3–38.7)	<0.001 *
Primary	438	19.3 (17.0–21.9)	209	18.4 (15.3–21.9)		229	20.9 (17.8–24.5)	
Secondary	871	14.6 (13.1–16.2)	527	14.4 (12.6–16.4)		344	15.4 (12.9–18.2)	
Tertiary	517	15.8 (13.6–18.3)	411	16.2 (13.7–19.1)		106	13.7 (10.4–18.0)	
**Marital status**								
Single	358	13.6 (11.5–16.0)	246	13.8 (11.3–16.7)	0.060	112	12.9 (9.3–17.6)	0.010 *
Married	1326	16.6 (15.1–18.1)	794	15.9 (14.3–17.7)		532	18.6 (16.0–12.5)	
Widow(er)/Divorcee/Separated	262	20.7 (17.3–24.7)	147	19.5 (15.3–24.5)		115	23.9 (18.8–29.9)	
***Enabling***								
**Employment status**								
Government	268	17.9 (14.6–21.8)	204	17.5 (13.6–22.1)	0.745	64	19.5 (14.2–26.2)	0.558
Private	480	15.0 (12.8–17.4)	334	14.9 (12.6–17.6)		146	15.3 (11.0–20.8)	
Self-employed	335	16.3 (14.1–18.9)	165	15.9 (13.1–19.3)		170	17.2 (14.0–20.9)	
Unemployed	863	16.5 (14.8–18.3)	484	15.8 (13.7–18.1)		379	18.4 (15.7–21.4)	
**Household income quintile**								
Q1 (20% poorest)	447	17.3 (15.2–19.6)	204	14.7 (12.1–17.9)	0.433	243	21.5 (18.4–25.1)	0.060
Q2	362	17.1 (14.7–19.8)	195	17.0 (14.0–20.6)		167	17.2 (13.7–21.4)	
Q3	378	16.4 (14.1–19.0)	218	16.4 (13.6–19.6)		160	16.5 (12.7–21.0)	
Q4	363	15.7 (13.1–18.6)	254	16.5 (13.6–19.9)		109	11.5 (8.4–15.5)	
Q5 (20% richest)	396	14.1 (11.8–16.7)	316	13.9 (11.5–16.6)		80	15.9 (8.8–26.9)	
**Covered by any healthcare coverage**								
Yes	1084	16.4 (14.7–18.3)	759	16.0 (14.1–18.1)	0.463	325	18.7 (15.2–22.9)	0.348
No	862	15.6 (14.2–17.2)	428	15.0 (13.1–17.2)		434	16.9 (14.9–19.1)	
***Health need***								
**Self-rated health**								
Excellent & Good	1099	12.3 (11.0–13.6)	685	12.0 (10.6–13.6)	<0.001 *	414	13.2 (11.0–15.7)	<0.001 *
Fair	733	28.0 (25.4–30.8)	430	27.8 (24.4–31.5)		303	28.5 (24.7–32.7)	
Poor & Very Poor	114	47.5 (38.8–56.4)	72	49.3 (38.8–59.9)		42	41.9 (28.4–56.7)	
**Presence of at least one long-term condition**								
Yes	728	21.3 (19.0–23.7)	417	20.4 (17.7–23.5)	<0.001 *	311	23.7 (20.1–27.7)	<0.001 *
No	1218	14.4 (13.0–15.9)	770	14.1 (12.5–15.8)		448	15.5 (13.2–18.1)	
***Health-seeking behavior***								
**Sought treatment from healthcare practitioner**								
Yes	1122	57.3 (53.7–60.8)	681	58.9 (24.3–63.3)	-	441	52.6 (47.6–57.6)	-
No	824	42.7 (39.2–46.3)	506	41.1 (36.7–45.7)		318	47.4 (42.4–52.4)	
**Self-medicated**								
Yes	438	23.3 (20.2–26.8)	258	23.2 (19.3–27.7)	-	180	23.6 (19.3–28.4)	-
No	1508	76.7 (73.2–79.8)	929	76.8 (72.3–80.7)		579	76.4 (71.6–80.7)	

*n*, count; %, percentage; CI, Confidence Interval; Q, Quintile. *p*-values were obtained by chi-square test; * indicates a statistical significance. Healthcare coverage refers to government employees’ health benefits, pensioner card, government-specific health fund, personal health insurance, employer-sponsored insurance, and panel clinic. Long-term condition refers to presence of any non-communicable diseases (diabetes, hypertension or hypercholesterolemia).

**Table 3:** Logistic regression model for health-seeking behaviors among Malaysian adults who reported sickness, stratified by urban-rural locality.

Factors	Sought Treatment from Healthcare Practitioner				Self-Medicated			
	Model I—Urban		Model II—Rural		Model III—Urban		Model IV—Rural	
	Crude OR (95% CI)	Adjusted OR (95% CI)	Crude OR (95% CI)	Adjusted OR (95% CI)	Crude OR (95% CI)	Adjusted OR (95% CI)	Crude OR (95% CI)	Adjusted OR (95% CI)
**Sex**								
Male	1.00 (ref)	1.00 (ref)	1.00 (ref)		1.00 (ref)		1.00 (ref)	
Female	1.35 (0.96–1.91)	1.33 (0.92–1.92)	0.98 (0.59–1.63)		1.10 (0.73–1.66)		0.98 (0.59–1.62)	
**Ethnicity**								
Malay	1.00 (ref)		1.00 (ref)		1.00 (ref)		1.00 (ref)	1.00 (ref)
Non-Malay	0.81 (0.54–1.20)		0.80 (0.52–1.25)		1.20 (0.76–1.88)		0.67 (0.39–1.14)	0.66 (0.38–1.13)
**Age (years)**								
18–34	1.00 (ref)		1.00 (ref)	1.00 (ref)	1.00 (ref)		1.00 (ref)	1.00 (ref)
35–59	1.26 (0.90–1.77)		0.90 (0.54–1.52)	0.71 (0.41–1.22)	0.90 (0.56–1.44)		1.57 (0.89–2.77)	1.58 (0.90–2.79)
60+	1.20 (0.72–1.97)		1.57 (0.82–3.00)	0.88 (0.42–1.87)	0.93 (0.52–1.65)		0.94 (0.52–1.69)	0.93 (0.52–1.66)
**Education level**								
No formal	1.53 (0.66–3.55)		0.98 (0.45–2.14)		3.69 (1.51–9.03)**	4.29 (1.81–10.17)**	0.73 (0.34–1.57)	
Primary	1.19 (0.81–1.75)		1.24 (0.71–2.15)		1.46 (0.87–2.46)	1.58 (0.93–2.66)	0.94 (0.50–1.79)	
Secondary	1.00 (ref)		1.00 (ref)		1.00 (ref)	1.00 (ref)	1.00 (ref)	
Tertiary	1.29 (0.84–1.98)		1.18 (0.58–2.43)		1.29 (0.78–2.15)	1.26 (0.76–2.09)	0.64 (0.28–1.47)	
**Marital status**								
Single	1.03 (0.68–1.56)		1.02 (0.52–2.00)		1.16 (0.71–1.89)		1.42 (0.77–2.60)	
Married	1.00 (ref)		1.00 (ref)		1.00 (ref)		1.00 (ref)	
Widow(er)/Divorcee/Separated	1.01 (0.54–1.92)		1.33 (0.64–2.73)		1.03 (0.58–1.84)		1.19 (0.56–2.52)	
**Employment status**								
Government	1.92 (1.07–3.43) *	1.82 (1.01–3.27) *	0.83 (0.40–1.71)		0.98 (0.45–2.12)		0.59 (0.20–1.72)	
Private	1.29 (0.80–2.09)	1.34 (0.84–2.16)	0.92 (0.45–1.91)		1.13 (0.63–2.05)		0.50 (0.23–1.11)	
Self-employed	1.00 (ref)	1.00 (ref)	1.00 (ref)		1.00 (ref)		1.00 (ref)	
Unemployed	1.59 (1.05–2.41)	1.31 (0.83–2.05)	1.24 (0.72–2.14)		1.02 (0.60–1.74)		0.66 (0.32–1.37)	
**Household income quintile**								
Q1 (20% poorest)	1.01 (0.58–1.77)		1.88 (0.78–4.56)		1.03 (0.55–1.94)		1.43 (0.35–5.78)	
Q2	0.76 (0.46–1.27)		1.70 (0.58–5.03)		1.24 (0.68–2.26)		1.45 (0.37–5.58)	
Q3	0.77 (0.44–1.33)		1.50 (0.61–3.70)		1.27 (0.65–2.47)		1.93 (0.50–7.47)	
Q4	0.60 (0.33–1.06)		1.96 (0.63–6.05)		1.34 (0.69–2.60)		1.74 (0.41–7.32)	
Q5 (20% richest)	1.00 (ref)		1.00 (ref)		1.00 (ref)		1.00 (ref)	
**Covered by any healthcare coverage**								
Yes	0.99 (0.70–1.40)		0.69 (0.44–1.07)	0.77 (0.47–1.27)	1.13 (0.75–1.72)		0.87 (0.49–1.54)	
No	1.00 (ref)		1.00 (ref)	1.00 (ref)	1.00 (ref)		1.00 (ref)	
**Self-rated health**								
Excellent & Good	1.00 (ref)	1.00 (ref)	1.00 (ref)	1.00 (ref)	1.00 (ref)	1.00 (ref)	1.00 (ref)	
Fair	1.51 (1.09–2.09) *	1.41 (0.98–2.04)	1.10 (0.69–1.75)	0.88 (0.54–1.43)	0.97 (0.66–1.42)	0.89 (0.62–1.29)	0.96 (0.55–1.69)	
Poor & Very Poor	3.04 (1.56–5.90) **	2.94 (1.47–5.88) ***	4.69 (1.69–13.06)**	3.68 (1.36–9.97)*	0.49 (0.21–1.13)	0.40 (0.16–0.98)	0.89 (0.31–2.50)	
**Presence of at least one long-term condition**								
Yes	1.28 (0.79–2.07)	1.27 (0.81–2.01)	2.01 (1.31–3.10)**	2.06 (1.23–3.45) **	0.90 (0.60–1.36)		0.82 (0.49–1.36)	
No	1.00 (ref)	1.00 (ref)	1.00 (ref)	1.00 (ref)	1.00 (ref)		1.00 (ref)	

Model I assessed the factors associated with seeking treatment among self-reported sick adults in urban locality; Model II assessed the factors associated with seeking treatment among self-reported sick adults in rural locality; Model III assessed the factors associated with self-medication among self-reported sick adults in urban locality; Model IV assessed the factors associated with self-medication among self-reported sick adults in rural locality. COR, Crude Odd Ratios; AOR, Adjusted Odd Ratios; CI, Confidence Interval; ref, reference category; Q, Quintile; * *p* < 0.05; ** *p* < 0.01; *** *p* < 0.001. Goodness of fit for model: Hosmer–Lemeshow statistic: Model I = 0.317; Model II = 0.801; Model III = 1.00; Model IV = 1.00. Healthcare coverage refers to government employees’ health benefits, pensioner card, government-specific health fund, personal health insurance, employer-sponsored insurance, and panel clinic.

## Data Availability

To protect the privacy of the respondents, the data set that supports the findings of this article is not publicly available. Request for data can be obtained from the Head of Centre for Biostatistics & Data Repository, National Institutes of Health, Ministry of Health Malaysia on reasonable request and with the permission from the Director General of Health, Malaysia.

## Supplementary Materials

The following are available online at https://www.mdpi.com/article/10.3390/ijerph19063193/s1, Table S1: Logistic regression model for self-rated health as poor to very poor among Malaysian adults who reported sickness.
