# Supplementary material for: The Health-Seeking Behavior among Malaysian Adults in Urban and Rural Areas Who Reported Sickness: Findings from the National Health and Morbidity Survey (NHMS) 2019
Source: Int J Environ Res Public Health. 2022 Mar 8;19(6):3193. doi: 10.3390/ijerph19063193 (PMC8954644; doi:10.3390/ijerph19063193)
Supplement: Supplementary file 1 [file ijerph-19-03193-s001.zip › ijerph-1519196-supplementary.pdf]

Table S1. Logistic regression model for self-rated health as poor to very poor among Malaysian adults who reported sickness

Factors	Crude OR (95% CI)	Adjusted OR (95% CI)
**Sex**		
Male	1.00 (ref)	
Female	0.77 (0.48 - 1.23)	
**Ethnicity**		
Malay	1.00 (ref)	
Non-Malay	1.24 (0.70 – 2.19)	
**Age (years)**		
18-34	1.00 (ref)	1.00 (ref)
35-59	1.76 (0.90 – 3.42)	1.13 (0.46 – 2.82)
60+	3.08 (1.52 – 6.25)*	0.90 (0.34 – 2.33)
**Education level**		
No formal	2.81 (1.30 – 6.07)**	1.86 (0.75 – 4.57)
Primary	2.06 (1.16 – 3.65)*	1.57 (0.86 – 2.84)
Secondary	1.00 (ref)	1.00 (ref)
Tertiary	0.51 (0.22 - 1.17)	0.71 (0.29 – 1.74)
**Marital status**		
Single	0.76 (0.40 - 1.45)	1.40 (0.65 – 3.02)
Married	1.00 (ref)	1.00 (ref)
Widow(er)/Divorcee/Separated	1.77 (0.98 – 3.21)	0.99 (0.48 – 2.03)
**Employment status**		
Government	0.45 (0.09 – 2.27)	0.65 (0.13 - 3.31)
Private	0.67 (0.25 – 1.77)	0.83 (0.27 - 2.51)
Self-employed	1.00 (ref)	1.00 (ref)
Unemployed	2.11 (1.02 – 4.40)*	1.87 (0.90 – 3.86)
**Household income quintile**		
Q1 (20% poorest)	3.90 (1.60 – 9.50)**	1.65 (0.65 – 4.18)
Q2	2.44 (0.94 – 6.34)	1.51 (0.56 – 4.10)
Q3	1.63 (0.63 – 4.15)	1.30 (0.52 – 3.25)
Q4	2.10 (0.75 – 5.90)	1.89 (0.64 – 5.09)
Q5 (20% richest)	1.00 (ref)	1.00 (ref)
**Covered by any healthcare coverage**		
Yes	0.62 (0.36 - 1.08)	1.13 (0.46. 2.82)
No	1.00 (ref)	0.90 (0.35 – 2.33)
**Presence of at least one long-term condition**		
Yes	2.93 (1.78 – 4.84)***	2.30 (1.19 – 4.43)*
No	1.00 (ref)	1.00 (ref)

COR, Crude Odd Ratios; AOR, Adjusted Odd Ratios; CI, Confidence Interval; ref, reference category; Q, Quintile; *p<0.05; **p<0.01; ***p<0.001.
Healthcare coverage refers to government employees' health benefits, pensioner card, government-specific health fund, personal health insurance, employer-sponsored insurance, and panel clinic.
